# QSAR Models for CXCR2 Receptor Antagonists Based on the Genetic Algorithm for Data Preprocessing Prior to Application of the PLS Linear Regression Method and Design of the New Compounds Using *In Silico* Virtual Screening

**DOI:** 10.3390/molecules16031928

**Published:** 2011-02-25

**Authors:** Tahereh Asadollahi, Shayessteh Dadfarnia, Ali Mohammad Haji Shabani, Jahan B. Ghasemi, Maryam Sarkhosh

**Affiliations:** 1 Department of Chemistry, Faculty of Science, Yazd University, Yazd 89195, Iran; 2 Department of Chemistry, Faculty of Science, K. N. Toosi University of Technology, Tehran, Iran

**Keywords:** QSAR, CXCR2 receptor, in silico screening, estimation of pIC_50_

## Abstract

The CXCR2 receptors play a pivotal role in inflammatory disorders and CXCR2 receptor antagonists can in principle be used in the treatment of inflammatory and related diseases. In this study, quantitative relationships between the structures of 130 antagonists of the CXCR2 receptors and their activities were investigated by the partial least squares (PLS) method. The genetic algorithm (GA) has been proposed for improvement of the performance of the PLS modeling by choosing the most relevant descriptors. The results of the factor analysis show that eight latent variables are able to describe about 86.77% of the variance in the experimental activity of the molecules in the training set. Power prediction of the QSAR models developed with SMLR, PLS and GA-PLS methods were evaluated using cross-validation, and validation through an external prediction set. The results showed satisfactory goodness-of-fit, robustness and perfect external predictive performance. A comparison between the different developed methods indicates that GA-PLS can be chosen as supreme model due to its better prediction ability than the other two methods. The applicability domain was used to define the area of reliable predictions. Furthermore, the *in silico* screening technique was applied to the proposed QSAR model and the structure and potency of new compounds were predicted. The developed models were found to be useful for the estimation of pIC_50_ of CXCR2 receptors for which no experimental data is available.

## 1. Introduction

The chemokine CXCR2 receptor, a seven-transmembrane G-protein-coupled receptor, was cloned and identified in the early 1990s [1,2,3]. Chemokines play the key roles in inflammation, wound healing, hematopoiesis and metastasis. The chemokines comprise a large protein family that can be divided into subfamilies on the bases of structural motifs. Chemokines mediate their biological effects via interaction with a large family of 7-transmembrane G Protein-coupled receptors. These receptors are divided into four subgroups: CC, C, CX_3_C and CXC chemokine ligands (where X represents an amino acid) depending upon the position of the N-terminal cysteine residues within the protein. The chemokine receptors CXCR2/CXCR1 were cloned and identified and are activated by IL-8 (CXCL8) [4,5]. Interleukin 8 (IL-8, CXCL8) and growth related oncogene α (GRO-α) are members of the CXC chemokine subfamily and have a role in the activation and recruitment of the neutrophils to the sites of the inflammation mediated through the CXCR2 receptor. When CXCL8 interacts with the CXCR2 and CXCR1 on the neutrophils, an intercellular response occurs, including calcium flux, degranulation and subsequently chemotaxis. Elevated levels of CXCL8 have been observed in the diseases such as arthritis and chronic obstructive pulmonary disease (COPD) [6]. In the light of these findings, small molecule antagonists of the CXCR2 receptor are attractive biological targets for molecular drug discovery [7].

During the past decades, different approaches have been used for the development of QSAR models. The major differences between these approaches are in the structural parameters (descriptors) used to characterize molecules and/or in the mathematical methods used to establish a correlation between the descriptor values and the biological activities. One of the most successful approaches for the prediction of the chemical properties based on the molecular structural information is modeling of quantitative structure-activity/property relationships (QSAR/QSPR). The main goal of QSAR/QSPR is to predict complex physical, chemical and biological properties of the compounds from molecular structures [8,9]. The close relationship which exists between bulk properties of the compounds and their molecular structures allows one to provide a clear connection between the macroscopic and the microscopic properties of matter. QSAR methodologies have the potential of decreasing substantially the time and effort required for the discovery of the new medicines or improvement of the efficiency of the current one. The success of the QSAR approach can be explained by the insights offered for the structural determination of chemical properties, and the possibility of estimating the properties of the new chemical compounds without any need for them to be synthesized and tested. However, the success of any QSAR model depends on the accuracy of input data, selection of the appropriate descriptors, statistical tools, and most importantly validation of the developed model [10,11,12,13]. A major step in constructing the QSAR models is to find a set of molecular descriptors that represents variation in the structural properties of the molecules.

QSAR analysis employs statistical methods to drive quantitative mathematical relationships between chemical structure and biological activity. Thus, the use of the QSAR for the development of a theoretical model for calculation of the IC_50_ (the half maximal inhibitory concentration) of a diverse set of compounds seems to be interesting. 

The strategy used in the QSAR models includes the following steps; (1) selection of a data set; (2) generation of the data molecular structures; (3) optimization of the geometry of the molecular structures by appropriate method; (4) generation of various structural descriptors; (5) application of variable selection or/and data reduction methods on the calculated descriptors; (6) regression analysis; and finally (7) evaluation of the validity and predictability of the developed QSAR models.

In the past, QSAR models have been built in the general field of chemokine antagonists including CCR1 [14], CCR5 [15,16], CXCR3 [17], CXCR4 [18] and one group of CXCR2 [19,20]. In this work, linear methods such as SMLR, PLS and GA-PLS are used to find quantitative relationships between the structures of several classes of CXCR2 antagonists and their biological activities, and the results obtained by these methods are compared. Furthermore, *in silico* screening is adopted to the QSAR model in order to predict the structure of new potentially active compounds.

## 2. Data and Methods

### 2.1. Data Set

The biological and chemical data of 130 CXCR2 antagonists, taken from literatures were selected for QSAR study [19,21,22,23]. The data set were heterogeneous, and involved several main classes of CXCR2 antagonists including; *N,N’*-diphenylureas, nicotinamide *N*-oxides, quinoxalines, triazolethiols, acylsulfonamide carboxylic acid bioisosteres, *N*-linked sulfonylurea, and furyl-3,4-diamino-3-cyclobut-3-ene-1,2-dione. The general structure and biological activities of the CXCR2 antagonists are provided in **Table 1**, **Table 2**, **Table 3**, **Table 4**, **Table 5**, **Table 6** and **Table 7**.

**Figure 1:**
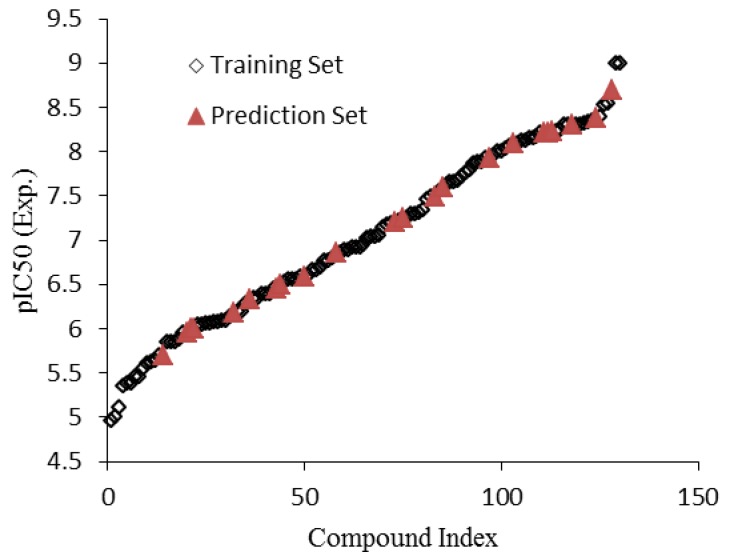
Distribution of pIC_50_ values for the whole data set.

In order to guarantee that training and prediction sets cover the total space occupied by the original data set, it was divided into two parts of training and predication set according to the Kennard-Stones algorithm [24]. The Kennard-Stones algorithm is known as one of the best ways of building training and prediction sets [25,26] and recently, it has been used in many QSAR studies [27,28]. Thus, the training set, which contains 108 compounds with pIC_50_s in the range of 4.96–9.00 was used for building up the QSAR model, whereas the prediction set containing 22 compounds (out of 130 compounds, *i.e.*, about 20% of the total number of compounds) with pIC_50_s in the range of 5.70–8.70 was used for evaluation of the model’s predictive ability. The distribution of pIC_50_ values of 130 essential CXCR2 antagonist receptors are demonstrated in **Figure 1**. As shown, these pIC_50_ values cover a wide range from 4.96 to 9.00.

**Figure 2:**
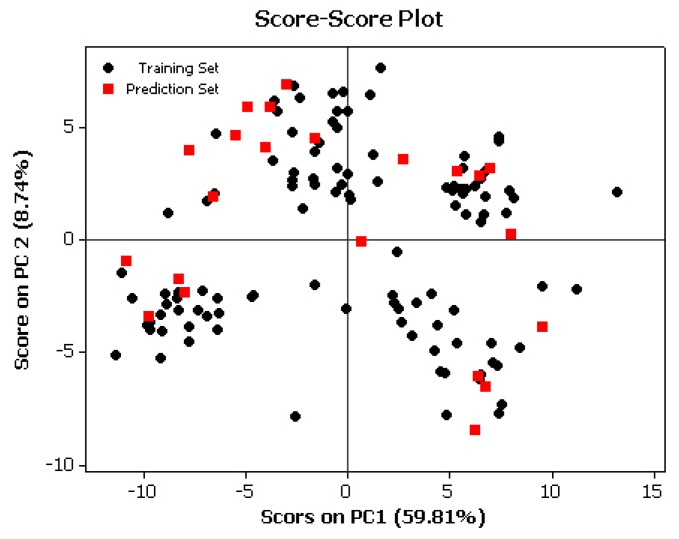
Score-Score plote.

Furthermore, in order to detect the homogeneities in the data set and to recognize the potential outliers in all of the molecules under study, the principal components analysis (PCA) [29] was performed with the calculated structural descriptors on the selected data set. **Figure 2** shows that with the two more significant PCs which explain 68.47% of the variation in the data set (59.86% by PC1 and 8.61% by PC2), the distribution of molecules over the region is homogeneous. Thus, the score plot is a reliable representation of the spatial distribution of the points for the data set.

### 2.2. Computer Hardware and Software

A Dell Personal Computer equipped with the Windows^®^ Vista operating system was used. HyperChem Release 7 software (Hypercube, Inc. Gainesville, Florida, USA 2002) was used to draw the molecular structures. Dragon software (Todeschini and Consonni, 2003 [30]) was employed for calculation of molecular structural descriptors. The selection of significant descriptors, which constructs a relationship between the biological activity of the data and its molecular structures, is an important step in QSAR modeling. For this purpose, the stepwise multiple linear regression method and genetic algorithm procedure were used to select the significant descriptors. The modeling was carried out using PLS Toolbox 3.5 (Eigen vector Research, Inc., Manson, WA, USA) as implemented in MATLAB. Other calculations were performed using MATLAB (version 7.5, Mathworks, Inc. Natick, MA, USA 2007) environment. 

### 2.3. Structural Descriptors

The theoretical molecular descriptors were derived from the chemical structure of the compounds. The 3D-structures of all the compounds were drawn using the HyperChem software. The resulting geometries were further refined by means of the semiempirical AM1 method and the molecular structures were optimized using the Polak-Rebiere algorithm until the root mean square gradient reached 0.1 kJ (mol Å). Then they were transferred into the Dragon program package (version 3) [30] to obtain the different molecular descriptors including constitutional, topological descriptors, RDF, 3D-Morse, and Geometrical descriptors [31]. Finally, the constant or near constant descriptors were omitted *i.e.*, one of the any two descriptors with an inter-correlation greater than 0.95 was removed to reduce the redundant and useless information.

### 2.4. Model Validation

Evaluation of a model’s stability and predictive ability is another key step in QSAR modeling. Different statistical parameters have been used for the evaluation of the suitability of the developed models for prediction of the activity of the studied compounds [32] this include cross validation coefficient (Q^2^ or R^2^_cv_), relative error percent of prediction sets (REP_Pred_), the root mean square error of prediction (RMSEP), root mean square error of cross-validation (RMSECV), validation through an external prediction set and Y-randomization. However, it should be noted that a high Q^2^ does not necessarily mean a high predictability of the developed model [31]. In other word, the high value of Q^2^ is a necessary condition, but not sufficient for a developed model to have high predictability. 

In order to assess the predictive ability and to check the statistical significance of the developed models, the proposed models were applied to predict the values of pIC_50_ of an external set that was not used in the development of the model. The predictive powers of the developed regression models on the training set were evaluated by predicted values of the prediction set. These parameters are listed in **Table 8** and show the good statistical qualities and low precision errors of the assessments.

The REP is calculated according to the following equation:

(1) $$REP(\%)=100/\bar{y}{\left[\frac{1}{n}{\left(y_{i}-{\hat{y}}_{i}\right)}^{2}\right]}^{0.5}$$

where ŷ_i_, y_i_, $\bar{y}$ and *n* are the predicted value, the experimental value, the mean of the experimental value in the prediction set and the number of samples, respectively.

The root mean square error cross validation (RMSECV) is a frequently used measure of the differences between the predicted values by a model or an estimator and the actually observed values from the objects being modeled or estimated. The RMSECV is defined as follows:

(2) $$RMSECV=\sqrt{\frac{\sum_{i=1}^{n}{\left({\hat{y}}_{i}-y_{i}\right)}^{2}}{n}}$$

where ŷ_i_, *y_i_* and *n* are the prediction value, the measured value and the number of measurements, respectively. The RMSECV is a measure of a model’s ability to predict new samples. The RMSECV is calculated via a leave one out cross-validation, where each sample is left out of the model formulation and then is predicted. The RMSEP is defined as a measure of the average difference between the predicated and experimental values at the predication stage. The RMSEP is calculated by applying Eq. (2) to the predication set. 

Most QSAR modeling methods implement the leave-one-out (LOO) or leave-some-out (LSO) cross-validation procedure [13]. The outcome from the cross-validation procedure is evaluated by cross-validation coefficient (Q^2^ or R^2^_CV_) which is used as the criteria of both robustness and the predictive ability of the model. Cross-validated coefficient of R^2^_CV_ (LOO-Q^2^) is calculated according to the following formula:

(3) $$Q_{ext}^{2}=1-\frac{\sum_{i=1}^{\mathrm{Pr}ed}{\left(y_{i}-{\hat{y}}_{i}\right)}^{2}}{\sum_{i-1}^{\mathrm{Pr}ed}{\left(y_{i}-{\bar{y}}_{tr}\right)}^{2}}\;$$

where ŷ_i_ and y_i_ are the predicted value, the experimental value (over the prediction set), respectively, and ${\bar{y}}_{tr}$ is the averaged value of the dependent variable for the training set. Tropsha used the following criteria for the external validation on the prediction set:

Q^2^ > 0.5

R^2^ > 0.6

0.85 < k < 1.15 or 0.85 < k’ < 1.15

(4) $$\frac{\left(R^{2}-R_{o}^{2}\right)}{R^{2}}or\frac{\left(R^{2}-R_{o}^{\prime 2}\right)}{R^{2}}\leq 0.1\;$$

(5) $$K=\frac{\sum_{i=1}^{ntest}y_{i}{\hat{y}}_{i}}{\sum_{i=1}^{ntest}{\hat{y}}_{i}^{2}}K^{\prime }=\frac{\sum_{i=1}^{ntest}y_{i}{\hat{y}}_{i}}{\sum_{i=1}^{ntest}y_{i}^{2}}\;$$

(6) $$R^{o}=1-\frac{\sum_{i=1}^{ntest}{\left({\hat{y}}_{i}-y_{i}^{ro}\right)}^{2}}{\sum_{i=1}^{ntest}{\left({\hat{y}}_{i}-{\bar{\hat{y}}}_{i}\right)}^{2}}\;\mathrm{where}\;y_{i}^{ro}=K{\hat{y}}_{i}$$

(7) $${R^{\prime }}^{o}=1-\frac{\sum_{i=1}^{ntest}{\left(y_{i}-{\hat{y}}_{i}^{ro}\right)}^{2}}{\sum_{i=1}^{ntest}{\left(y_{i}-{\bar{y}}_{i}\right)}^{2}}\;\mathrm{where}\;{\hat{y}}_{i}^{ro}=K^{\prime }y_{i}$$

In these equations, *R*^2^ is the correlation coefficient of regression between the experimental values and the prediction activities of the compounds on the training and prediction sets. R^2^_o_, R’^2^_o,_ are mathematically defined as the regression of the experimental activities against predicted activities and regression of the predicted activities against experimental activities, respectively; where as, k and k’ are the slopes of these equations [33]. When these criteria are satisfied, it can be said that the model is predictive.

Furthermore, in order to assess the robustness of the model, the Y-randomization test was applied. The dependent variable vector (inhibitory activity) was randomly shuffled and a new QSAR model was developed using the original independent variable matrix. As was expected the new QSAR models (after several repetitions) have low *R*^2^ and *Q*^2^ values; the results are shown in **Table 9**.

## 3. Results and Discussion

The predictive ability of QSAR/QSPR models is affected by two factors: the descriptors, which must carry enough of the molecular structure information for the interpretation of the activity/property; and the employed modeling method. However, with too many descriptors, there is the possibility of over fitting of the statistical methods. Thus, in QSAR/QSPR studies the identification and selection of descriptors which provide maximum information in activity variations and have minimum co-linearity is important. On the other hand, the use of PLS usually results in well fitted stable models which have high predictive ability, but the estimation is not always very accurate and stable over the time. Therefore, a genetic algorithm (GA) [34] with a PLS regression improves the model accuracy in the selection of proper descriptors.

### 3.1. Stepwise Multiple Linear Regression (MLR)

**Figure 3:**
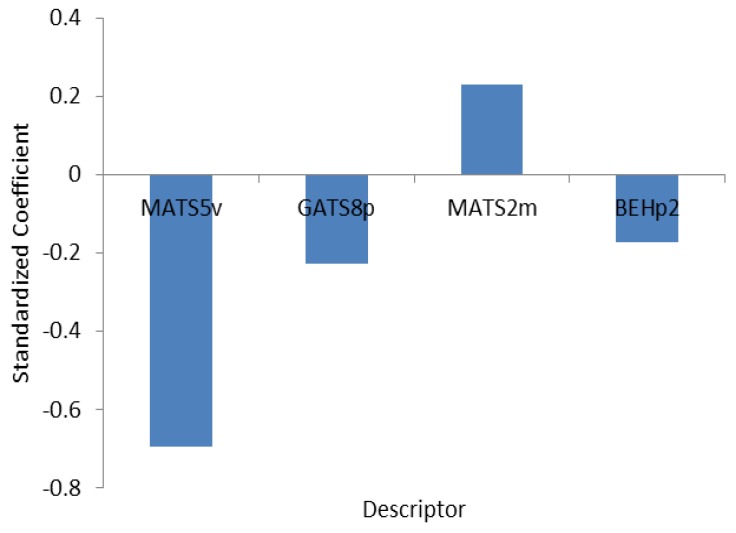
Standardized coefficients versus descriptors in MLR model.

On the basis of Kennard-Stones algorithm, 108 compounds out of 130 were selected as the training set and the remaining 22 were selected as the test set. Stepwise regression was used on the training data set to select the significant descriptors and it was found that between 733 calculated descriptors the MATS5v (Moran autocorrelation-lag5/weighted by atomic van der Waals volumes), GATS8P (Moran autocorrelation-lag8/weighted by atomic polarizabilites), MATS2m (Moran autocorrelation-lag2/weighted by atomic masses) and BEHp2 (highest eigenvalue n. 2 of burden matrix/weighted by atomic polarizabilites) construct the best model and there was no significant correlation between these descriptors (**Table 10**). So, they were selected for the further study. The selected physicochemical descriptors serve as the first guideline for the design of novel and the potent antagonists of CXCR2. The selected parameters used for development of the QSAR model are listed in **Table 11**. The model was produced by applying the multiple linear regression (MLR) technique on a database containing the training set. The relative importance and contribution of each descriptor in the model was determined by the calculation of the value of the mean effect (MF) [35] for each descriptor using the following equation:

(8) $$MF=\frac{\beta_{j}\sum_{i=1}^{i-n}d_{ij}}{\sum_{j}^{m}\beta_{j}\sum_{i}^{n}d_{ij}}$$

where MF*j* represents the mean effect for the descriptor *j, βj* is the coefficient of the descriptor *j*, *dij* is the value of the interested descriptors for each molecule and *m* is the number of descriptors in the model. The MF value shows the relative importance of each descriptor in compare to the other descriptors. The MF of the descriptor MATS5v, GATS8p, MATS2m and BEHp2 are also shown in **Table 11** and indicate that among the selected descriptors, the most important one is MATS2m (Moran autocorrelation-lag2/weighted by atomic masses) as it has the highest mean effect value and has the largest effect on the pIC_50_ of the compound. The effect of MATS5v, GATS8p, MATS2m and BEHp2 for the QSAR study of CXCR2 receptors and the standardized regression coefficient on the significance of an individual descriptor in the model is shown in Figure 3 and indicates that, the greater the absolute value of a coefficient, the greater the weight of the variable in the model.

Using the descriptors selected by the stepwise regression method, a new MLR equation was developed on the basis of the training set:

pIC_50_ = −8.92 − 5.41MATS5v − 1.34GATS8p + 31.53MATS2m − 3.54BEHp2

n = 122, R^2^ = 0.78, Q^2^ = 0.66, F = 51.2

where n and F are the compound’s number and the *F*-ratio, respectively.

**Figure 4:**
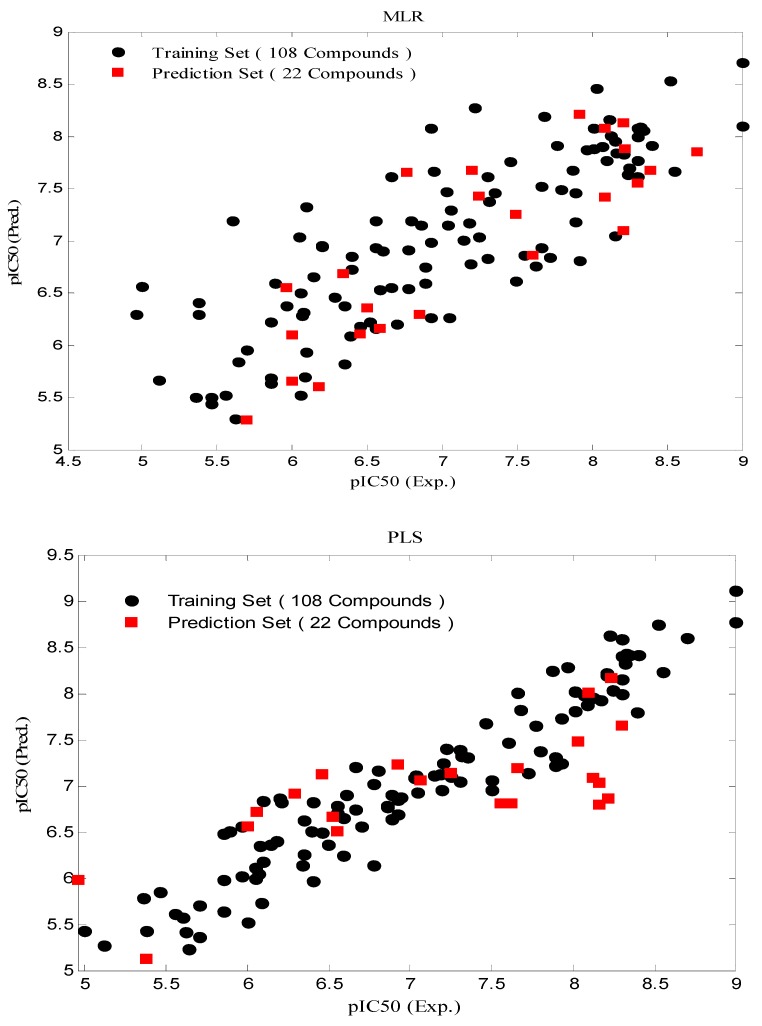

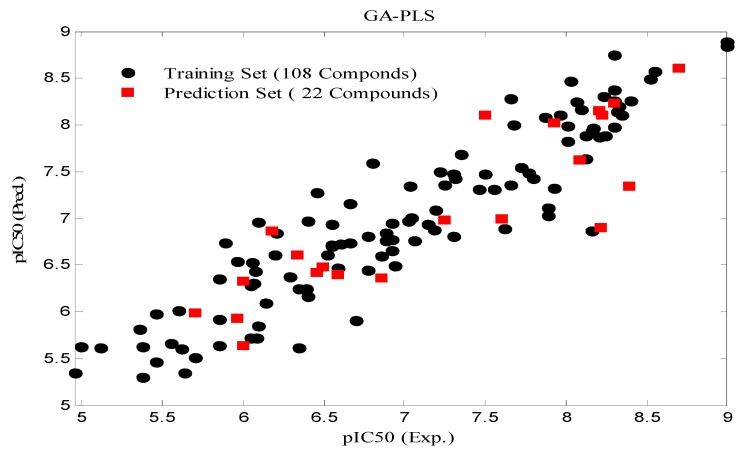
Predicted pIC_50_ values by (a) MLR; (b) PLS and (c) GA-PLS modeling *vs.* experimental pIC_50_ values.

In the further study, the constructed model from the training set was used to evaluate the predictive ability of the produced model by predicting the pIC_50_ values in the prediction set. The results are given in **Table 12** and **Figure 4**.

### 3.2. Interpretation of the Selected Descriptors

The binding of a ligand to a target depends on the shape of the ligand and on a variety of factors such as molecular electrostatic potential, polarizability, hydrophobicity, and lipophobicity. Therefore, in a QSAR study the strategy for encoding molecular information, either explicitly or implicitly, should account for these physicochemical effects. Furthermore, since the data sets usually include molecules of different size with different numbers of atoms, the structural encoding schemes must allow comparison between such molecules. The descriptors, MATS5v, GATS8p and MATS2m are *Autocorrelation of Topological Structure.* The 2D-autocorrelation descriptors explain how the values of certain functions, at intervals equal to the *lag*, are correlated. The 2D autocorrelation descriptors represent the topological structure of the compounds, but are more complex in nature when compared to the classical topological descriptors. The computation of these descriptors involves the summations of different autocorrelation functions corresponding to different structural lags and leads to different autocorrelation vectors corresponding to the lengths of the sub-structural fragments. Basically, the pool of 2D autocorrelation descriptors defines a wide 2D space. On behalf of a greater applicability, physicochemical properties (atomic masses, atomic van der Waals volumes, atomic Sanderson electronegativities, and atomic polarizabilities) were inserted as weighting components. As a result, these descriptors address the topology of the structure or parts thereof in association with a specific physicochemical property. Bearing in mind this aspect, the interpretation of 2D autocorrelation descriptors was uneasy. 

*BCTU descriptors* were designed to encode atomic properties relevant to intermolecular interactions. The three standard BCUT descriptor types–atomic charge, polarizability and hydrogen bonding properties—that are relevant to intermolecular interactions are supported. The BCUT (Burden-CAS-University of Texas eigenvalues) descriptors are the eigenvalues of a modified connectivity matrix known as the Burden matrix [17]. The BCUT metrics are extensions of parameters originally developed by Burden. The Burden parameters are based on a combination of the atomic number for each atom and a description of the nominal bond-type for adjacent and nonadjacent atoms. Among the eigenvalues obtained from B matrix, the highest eigenvalues have been demonstrated to reflect the relevant aspects of molecular structure, and are therefore useful for similarity searching. By B eigenvalue decomposition, one can find the best structure for the molecules, e.g., number of atoms, number of bonds and the electronic distributions of the whole molecule. With respect to this concept, B eigenvalues may play a good role in the prediction in addition to *BEHp2*.

### 3.3. Partial Least Squares (PLS)

The general purpose of the linear regression method is to quantify the relationship between several independent or predictive variables and a dependent variable. Independent or predictive variables could be various physicochemical descriptors of the molecules, their principle components or latent variables. The partial least squares (PLS) method is used to establish relationships between the dependent variables of the Y matrix and the descriptors of the X matrix (as independent variables also called “latent” variables) [34]. The procedure performs a principle component analysis on the independent variables matrix and simultaneously maximizing the correlation with the dependent variables matrix. The number of appropriate latent variables (LVs) for describing the best developed model was found out by evaluating the root mean square error cross-validation (RMSECV) while the number of latent variables was changed.

**Figure 5:**
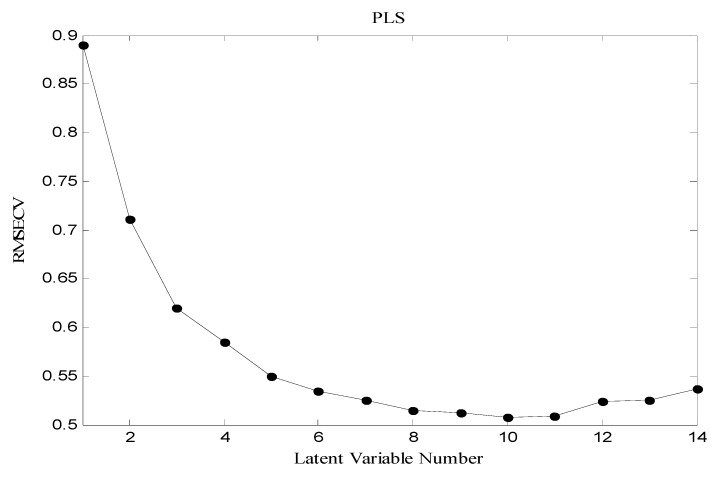

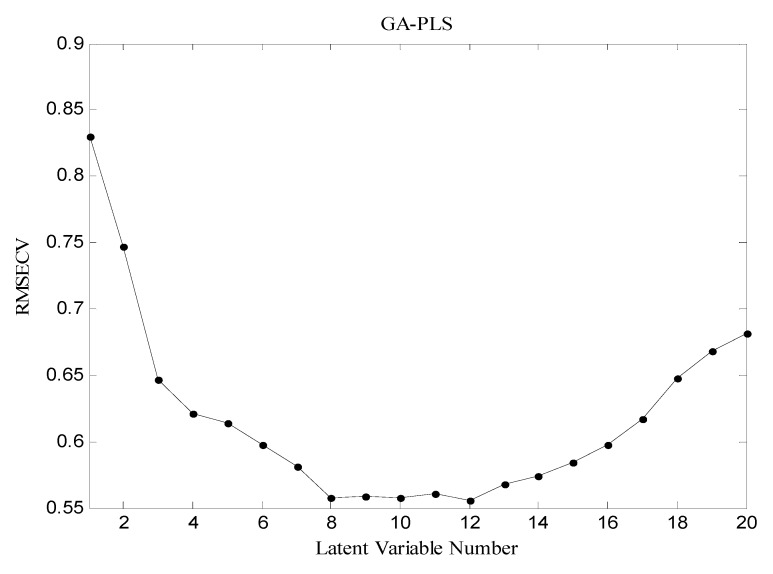
The RMSECV versus number of LVs.

As it is shown in **Figure 5** the RMSECV is minimized when the value of LVs is 7 and it is increased significantly when the numbers of LVs are greater than 11. Thus, the optimum LVs for the training set of PLS method was chosen to be 7. The developed PLS regression model with 7 LVs shows a high correlation between the experimental and predicted values of pIC_50_ in training set (*R*^2^ = 0.74 and RMSECV = 0.6).

Finally, for the evaluation of the predictive ability of the developed model, the Q^2^ value and the external validation method were performed. A high Q^2^ and *R*^2^ values (Q^2^ > 0.5) were considered as a proof of high predictive ability of the model. The external validation method was performed by dividing the original data set randomly into two parts, training and prediction set, and the values of pIC_50_ of molecules in the prediction set were predicted by the developed model. The results of the calculated *R*^2^, Q^2^, REP%, RMSECV and etc. for prediction set are reported in Table 2.

It should be noted that even when there is no correlation between the LOO- cross-validated R^2^ (Q^2^) and regression coefficient R^2^ for a predictive set with known values of biological activities, the validated model can be used for predicting activities/ properties of new chemicals [33,36]. Furthermore, As the results reveal, the PLS method is an efficient approach in monitoring many complex processes and is capable of strongly reducing cross-correlated data set with high dimension to a smaller and interpretable set of principle components or latent variables. 

### 3.4. Partial Least Squares combined with Genetic Algorithm (GA-PLS)

As mentioned before, one of the problems in choosing the set of molecular descriptors is the co-linearity within them. To overcome this problem some workers tried to combine the genetic algorithms (GA) with PLS [37,38,39]. GA-PLS consists of three basic steps. (1) Creation of an initial population of chromosomes in which each chromosome is a binary bit string by which the existence of a variable is represented; (2) Evaluation of fitness of each chromosome in the population by the internal predictivity of PLS. Thus, the squared predictive correlation coefficient (Q^2^) by the leave-one-out procedure in cross-validation is used as the internal predictivity [40]; (3) Reproduction of the population of chromosomes in the next generation. The operations of selection, cross-over and mutation of chromosomes, are made in this step. Then, steps 2 and 3 are continued until the number of the repetitions has reached the designated number of generations. The effective factors in the GA such as repetition rate, rate of mutation, number of chromosomes and generation are optimized.

Rogers and Hopfinger first applied GA-PLS method in QSAR analysis and stated that it is very effective and superior to PLS method. In this paper, to find the more convenient set of descriptors, a GA-PLS analysis was performed [41,42,43].

All descriptors were preprocessed by auto scaling before performing the GA-PLS was performed. The GA was optimized by variation and selection of the fitness values. The fitness function is defined as:

100−{[∑i=1n(yi−y^i)2/n][∑i=1n(yi−y¯i)2/k]}×100

where ${\hat{y}}_{i}$ is the predicted value of a sample i, n is the number of samples, k = n − 1 is the number of samples used in cross-validation. The definitions and types of selected descriptors are given in **Table 13**. The QSAR model was derived by the doing the GA analysis with partial least squares (PLS)-regression method for the population size of 64 and mutation rate of 0.003. Other parameters are summarized in **Table 14**. Results of *R*^2^, REP%, RMSEP and Q^2^ for prediction set of GA-PLS study are also reported in **Table 2** and as it is shown the results of this analysis are similar to those obtained by PLS method but the Q^2^ and *R*^2^ value of the GA-PLS were improved in compare to the MLR and PLS methods. However, the interpretations of the chemical properties of these descriptors are difficult as their definition is based on mathematics. The details are described in the handbook and literature of Dragon software [30]. Further more, although these results show that the GA method is a satisfactory correspondence for variable selection, but more experiments are needed to generalize the superiority of GA-PLS over other techniques.

### 3.5. In Silico Screening

The *in silico* screening procedure is a useful tool for predicting and identifying new biologically active compounds with improved characteristics prior to their actual synthesis [44,45]. Thus, the *in silico* procedure can be applied as a physico-chemical filter to reduce the number of compounds to be tested experimentally for hit/lead generation. In other words, the *in silico* procedure minimizes the time and cost associated with identifying new leads. A virtual screening was performed by insertion, deletion and substitution of different substitutes on the original molecules [46,47] and the effects of the structural modifications on the biological activity were investigated. Then, the domain of application of QSAR model was defined to use the model for screening new compounds. The applicability domain (AD) of QSAR model was used to verify the prediction reliability, to identify the problematic compounds and to predict the compounds with acceptable activity that falls within this domain. Several methods have been used for determination of the AD of QSAR models [48], but the most common one is described by Gramatica [49] which used the leverage values for each compound. The leverage approach allows the determination of the position of new chemical in the QSAR model; *i.e.*, whether a new chemical will lie within the structural model domain or outside of it. Furthermore, the leverage approach along with the Williams plot is used to determine the applicability domain in all QSAR models. 

To construct the William plot, the leverage *h_i_* for each chemical compound, in which QSAR model was used to predict its activity, was calculated according to the following equation:

$$h_{i}=x_{i}^{T}(X^{T}X)x_{i}$$

where *x_i_* is the descriptor vector of the considered compound and *X* is the descriptor matrix derived from the training set descriptor values and the warning leverage (h*) was determined as [48]:

$$h^{*}=\frac{3(p+1)}{n}$$

where *n* is the number of training compounds, *p* is the number of predictor variables. The defined applicability domain (AD) was then visualized via a Williams plot, the plot of the standardized residuals versus the leverage values (*h*). A compound with *h*_i_ > h^*^ seriously influences the regression performance and may be excluded from the applicability domain, but it doesn’t appear to be an outlier because its standardized residual may be small. Moreover, a value of 3 for standardized residuals is commonly used as a cut-off value for accepting predictions, because points that lie within ±3 standardized residual from the mean cover 99% of the normally distributed data [50]. Thus, the leverage and the standardized residual were combined for the characterization of the applicability domain. 

**Figure 6:**
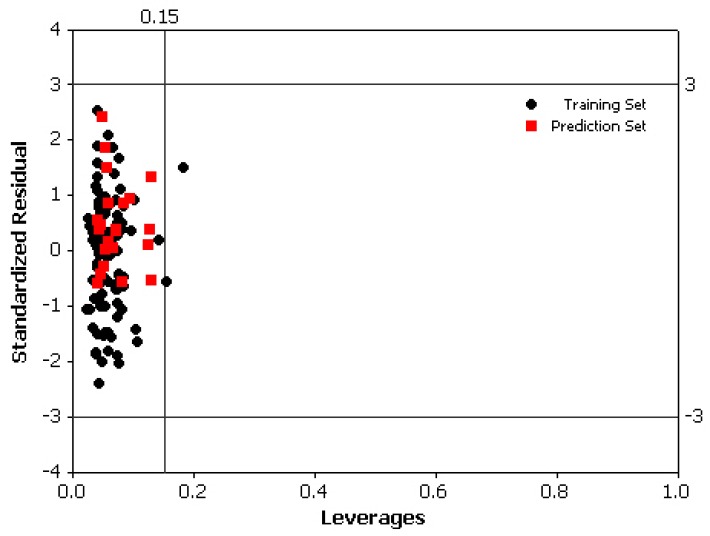
Williams plot of standardized residual versus leverage.

The Williams plot for the QSAR is illustrated in **Figure 6**. The warning leverage (h*), was found to be 0.25 for the developed QSAR model. The chemicals that had a standardized residual more than three times of the standard deviation units were considered to be outliers while chemicals with a leverage value higher than h* were considered to be influential or high leverage chemicals. Based on the leverages (*h* > 0.25), the one compound were found to be outside of the defined AD (**Figure 6**) of the QSAR model, so, it was identified as structurally influential chemical based on its large leverage value (*h* > h*).

Next, the *in silico* screening was applied to the design of new structures with potential CXCR2 inhibitors according to the developed QSAR model and was validated by the developed GA-PLS model. For this purpose, compound 66 of the N,N’-diphenylurea derivatives listed in **Table 1**, **Table 2**, **Table 3**, **Table 4**, **Table 5**, **Table 6** and **Table 7** (IC_50_ = 8.22) was selected as a template due to its good inhibition. The molecule was modified in such a way that its synthesis was experimentally possible. Then, the *in silico* screen was applied by substituting different groups in the X and Y positions of the ring; the results of this investigation are given in **Table 15**. The model tolerated various *N,N’*-diphenylurea substituents since all of the studied derivatives were within the applicability domain. Among different molecules designed, the compound **10c** showed the best activity (pIC_50_ = 8.50). Thus, in order to clarify the relation between the activities of the compounds with different functional group, this compound was selected for further structural modification. So, in the next step the oxygen of the amide group of compound **10c** was substituted by different function groups, the results are demonstrated in **Table 16**. As it is shown, the model tolerate all the compound designed on the bases of molecule **10c**, and the best predicted activity was found for the compound **9d** (where X = S). Thus, it is demonstrating that using a simple QSAR model, it is possible to simultaneously identify compounds with improved activity and to determine the structural modifications that don’t fall within the applicability domain. Finally, this result confirms the reliability of the models and it shows that with the construction of the QSAR model and use of *in silico* screening it is possible to identify new synthetic targets for drug discovery.

## 4. Conclusions

In this study, three different modeling methods, SMLR, PLS and GA-PLS were used in the construction of a QSAR model for CXCR2 antagonists and the resulting models were compared. It was shown that performing GA prior to the calibration, yields a regression model with improved predictive power. The accuracy and predictability of the proposed models were illustrated by various criteria, including cross-validation, relative error percent of prediction sets (REP_Pred_), the root mean square error of prediction (RMSEP), root mean square error of cross-validation (RMSECV), validation through and Y-randomization. It was also shown that the proposed method is a useful aid for reduction of the time and cost of synthesis and activity determination of CXCR2 receptor antagonists. Furthermore, the results confirm that among the construction models used, the GA-PLS is superior for prediction of the IC_50_ of CXCR2 antagonists. Our future work will focus on validation for putative CXCR2 antagonists for virtual screening. 

## Figures and Tables

**Table 1:**
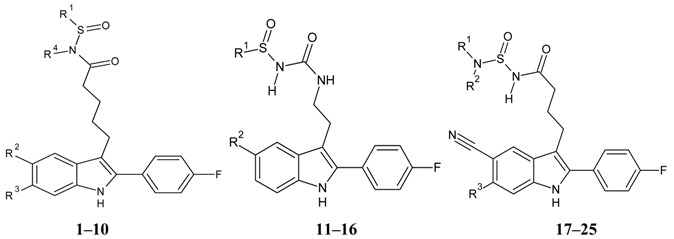
Structures and biological activities of the acylsulfonamide derivatives.

Compound	R^1^	R^2^	R^3^	R^4^	IC_50_ for CXCR2 (µM)	pIC_50_
1	Me	CN	H	H	0.07	7.14
2	Me	Br	H	H	0.17	6.77
3	Et	CN	H	H	0.06	7.19
4	n-Pr	CN	H	H	1.30	5.89
5	Bn	CN	H	H	1.40	5.85
6	i-Pr	CN	H	H	0.22	6.66
7	Ph	CN	H	H	0.26	6.58
8	CF_3_	CN	H	H	0.09	7.06
9	Me	CN	OMe	H	0.16	6.80
10	Me	CN	Me	H	0.02	7.72
11	Me	Br	-	-	0.25	6.60
12	Me	CN	-	-	0.64	6.19
13	Ph	Br	-	-	0.12	6.92
14	Ph	CN	-	-	0.14	6.85
15	*o*-Cl-Phenyl	CN	-	-	0.40	6.40
16	*p*-F-Phenyl	CN	-	-	0.52	6.28
17	Me	Me	H	-	0.05	7.30
18	Me	H	H	-	0.12	6.92
19	H	H	H	-	0.07	7.18
20	Et	Et	H	-	1.10	5.96
21	n-Butyl	H	H	-	1.10	5.96
22	Ph	H	H	-	0.88	6.05
23	-CH_2_CH_2_OMe	H	H	-	0.26	6.58
24	Me	Me	OMe	-	0.06	7.24
25	Me	Me	Me	-	0.02	7.62

**Table 2:**
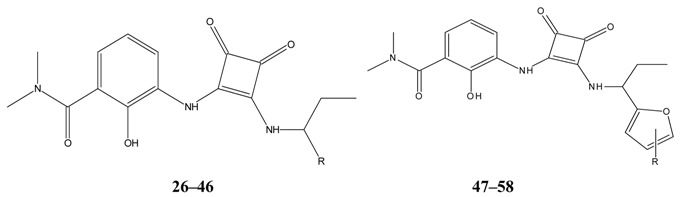
Structures and biological activities of the furyl and hetrocyclic-3,4-diamino-3-cyclobut-3-ene-1,2-dione derivatives.

Compound	R	IC_50_ CXCR2 (nM)	pIC_50_
26	5-H	0.005	8.3
27	5-Me	0.006	8.24
28	5-Et	0.004	8.39
29	5-Br	0.005	8.33
30	5-Cl	0.005	8.32
31	5-CF_3_	0.017	7.76
32	5-CF_2_H	0.007	8.17
33	5-CH_2_OH	0.003	8.55
34	5-CH_2_N(Me)_2_	0.094	7.03
35	5-CON(Me)_2_	0.171	6.77
36	5-(20Cl)Ph	0.049	7.31
37	5-(2-CF_3_)Ph	0.15	6.82
38	5-(3-Cl)Ph	0.058	7.24
39	5-(3-CF_3_)Ph	0.087	7.06
40	4-Cl	0.0045	8.35
41	4-Br	0.005	8.30
42	4-(4-Pyridyl)	0.009	8.02
43	4-(3-Thienyl)	0.008	8.09
44	4-(3,5-Dimethyl-4-isoxazoyl)	0.008	8.12
45	2,3-Benzofuran	0.003	8.46
46	3-Br	0.016	7.78
47	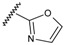	8.6	8.06
48	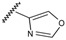	10.9	7.96
49	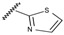	9.8	8.01
50	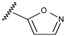	9.8	8.01
51	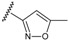	7.5	8.12
52	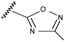	8.2	8.10
53	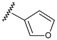	8.0	8.10
54	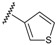	5.8	8.24
55	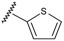	6.2	8.21
56	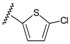	6.2	8.21
57	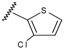	21	7.68
58	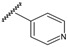	50	7.30

**Table 3:**
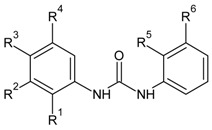
Structures and biological activities of the *N,N’*-diphenylureas derivatives.

Compound	R^1^	R^2^	R^3^	R^4^	R^5^	R^6^	IC_50_ for CXCR2 (nM)	pIC_50_
59	OH	H	Cl	H	Br	H	906	6.04
60	OH	Cl	Cl	H	Br	H	63	7.20
61	OH	CONH_2_	Cl	H	Br	H	10	8.00
62	OH	CH_2_NH_2_	Cl	H	Br	H	114	6.94
63	OH	SO_2_NH_2_	Cl	H	Br	H	7	8.15
64	OH	SO_2_NMe_2_	Cl	H	Br	H	12	7.92
65	OH	H	CN	H	Br	H	25	7.60
66	OH	Br	CN	H	Br	H	6	8.22
67	OH	Cl	CN	H	Br	H	22	7.66
68	OH	CN	Cl	H	Br	H	57	7.24
69	OH	H	NO_2_	H	Br	H	22	7.66
70	OH	H	NO_2_	H	H	H	320	6.49
71	OH	NO_2_	H	H	H	H	860	6.07
72	OH	H	H	NO_2_	H	H	10900	4.96
73	OH	H	CN	H	H	H	200	6.70
74	OH	SO_2_NH_2_	Cl	H	Cl	Cl	9.3	8.03
75	–N=N–NH–		CN	H	Br	H	39	7.49

**Table 4:**
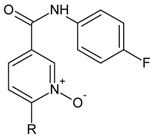
Structures and biological activities of the nikotinamide *N*-oxides derivatives.

Compound	R	IC_50_ for CXCR2 (nM)	pIC_50_
76	-SO_2_C_2_H5	130	6.87
77	-SO_2_CH(CH_3_)_2_	400	6.40
78	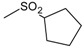	460	6.34
79	-SO_2_C_6_H_5_	90	7.05
80	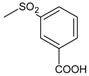	32	7.49
81	-SO_2_CH_2_C_6_H_5_	280	6.55
82	Cl	1000	6.00

**Table 5:**
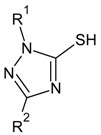
Structures and biological activities of the triazolethiol derivatives.

Compound	R^1^	R^2^	IC_50_ for CXCR2 (nM)	pIC_50_
83	C_6_H_5_CH_2_	C_6_H_5_	2400	5.62
84	3-OHC_6_H_4_CH_2_	C_6_H_5_	4400	5.36
85	C_6_H_5_CH_2_	4-Pyridinyl	7700	5.11
86	C_6_H_5_CH_2_	2-Furanyl	4200	5.38
87	C_6_H_5_CH_2_	4-CNC_6_H_4_	3500	5.46
88	C_6_H_5_CH_2_	3-CF_3_C_6_H_4_	3500	5.46
89	C_6_H_5_CH_2_	4-CF_3_C_6_H_4_	2800	5.55
90	C_6_H_5_CH_2_	4-CH_3_OC_6_H_4_	2300	5.64
91	C_6_H_5_CH_2_	3,5-diClC_6_H_3_	2000	5.70
92	C_6_H_5_CH_2_	2-Thienyl	2000	5.70
93	C_6_H_5_CH_2_	2-CH_3_C_6_H_4_	1400	5.85
94	C_6_H_5_CH_2_	2-CH_3_OC_6_H_4_	1400	5.85
95	C_6_H_5_CH_2_	3-ClC_6_H_4_	1000	6.00
96	C_6_H_5_CH_2_	2-FC_6_H_4_	890	6.05
97	C_6_H_5_CH_2_	4-ClC_6_H_4_	830	6.08
98	C_6_H_5_CH_2_	3,4-diClC_6_H_3_	800	6.10
99	C_6_H_5_CH_2_	2,5-diClC_6_H_3_	670	6.17
100	C_6_H_5_CH_2_	2-ClC_6_H_4_	450	6.35
101	C_6_H_5_CH_2_	2,4-diClC_6_H_3_	410	6.39
102	C_6_H_5_CH_2_	2-BrC_6_H_4_	350	6.46
103	C_6_H_5_CH_2_	2,3-diClC_6_H_3_	350	6.46
104	4- CH_3_OC_6_H_4_CH_2_	2,4-diClC_6_H_3_	10000	5.00
105	3-CH_3_OC_6_H_4_CH_2_	2,4-diClC_6_H_3_	4200	5.38
106	3-CH_3_C_6_H_4_CH_2_	2,4-diClC_6_H_3_	730	6.14
107	4-Cl C_6_H_4_CH_2_	2,4-diClC_6_H_3_	300	6.52
108	3-C_6_H_5_O C_6_H_4_CH_2_	2,4-diClC_6_H_3_	170	6.77
109	3-Cl C_6_H_4_CH_2_	2,4-diClC_6_H_3_	92	7.04
110	3-Cl C_6_H_4_CH_2_	2-ClC_6_H_4_	28	7.55

**Table 6:** Structures and biological activities of the bicyclic CXCR2 antagonists.

Compound		IC_50_ for CXCR2 (nM)	pIC_50_
111	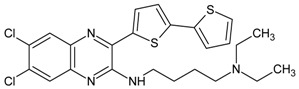	160	6.80
112	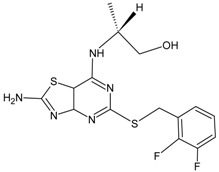	4	8.40
113	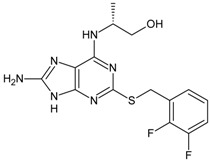	13	7.89
114	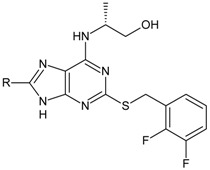	630	6.20
115	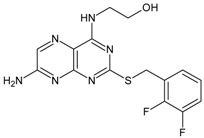	7	8.15
116	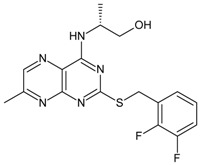	280	6.55
117	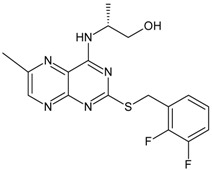	140	6.85
118	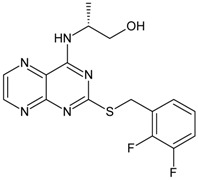	280	6.55
119	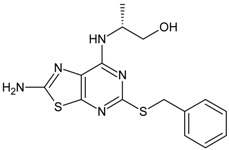	850	6.07
120	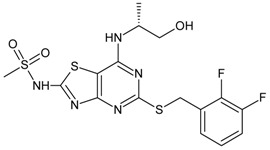	5	8.30
121	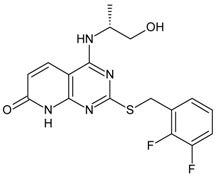	350	6.46
122	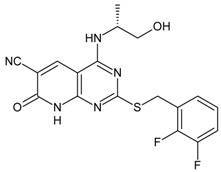	16	7.80
123	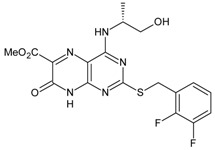	2	8.70
124	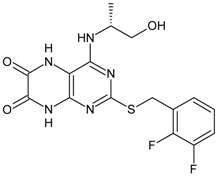	45	7.35
125	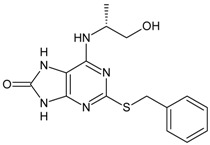	2500	5.60
126	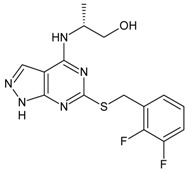	220	6.66

**Table 7:**
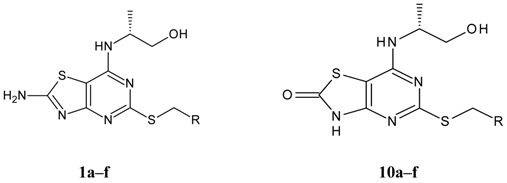
Structures and biological activities of the bicyclic CXCR2 antagonists.

Compound	R	IC_50_ for CXCR2 (nM)	pIC_50_
**1a** **10a**	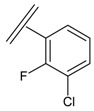	3 1	8.52 9
**1b** **10b**	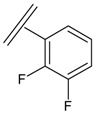	4 1	8.40 9.00
**1c** **10c**	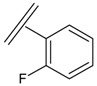	13 2	7.89 8.70
**1d** **10d**	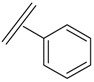	13 5	7.89 8.30
**1e** **10e**	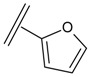	35 5	7.46 8.30
**1f** **10f**	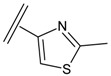	120 60	6.92 7.22

**Table 8:** Statistical parameters obtained by applying the PLS, GA-PLS and SMLR.

Parameter	PLS	GA-PLS	SMLR
RMSEP	0.50	0.51	0.56
ARE_Pred._	5.98	5.53	1.3
R^2^	0.748	0.779	0.78
R^2^_Training Set_	0.727	0.88	0.68
Q^2^	0.68	0.713	0.66
SEP	0.50	0.51	0.53
R^2^ − R_o_^2^/R^2^	−0.291	−0.254	−0.254
K	1.019	1.035	0.962

**Table 9:** R^2^ and Q^2^ values after several Y-randomization tests.

Iteration	PLS		GA-PLS	
	R^2^	Q^2^	R^2^	Q^2^
1	0.0047	−0.949	0.010	−0.577
2	0.005	−0.423	0.010	−0.919
3	0.039	−0.467	0.036	−0.417
4	0.12	−0.198	0.019	−0.506
5	0.005	−0.955	0.006	−0.878
6	0.005	−0.955	0.153	−0.063
7	0.006	−0.967	0.084	−0.245
8	0.186	−1.601	0.001	−0.699
9	0.002	−0.753	0.073	−1.21
10	0.171	−1.57	0.147	−0.41

**Table 10:** Correlation matrix for MLR model.

	pIC_50_	MATS5v	GATS8p	MATS2m	BEHp2
pIC_50_	1				
MATS5v	−0.26863	1			
GATS8P	−0.16055	−0.00856	1		
MATS2m	0.001149	−0.08958	−0.0286	1	
BEHp2	0.214723	−0.04342	−05904	0.000615	1

**Table 11:** Details of the constructed MLR model.

Descriptor^a^	Coefficient	MF^b^
MATS5v	−8.9918 (±8.729)	−0.254
GATS8P	−5.409 (±0.463)	−0.063
MATS2m	−1.337 (±0.349)	1.484
BEHp2	31.527 (±7.936)	−0.166
Constant	−3.539 (±1.156)	

^a^ The name and chemical meanings of descriptors are explained in the text; ^b^ MF refer to the mean effect value.

**Table 12:** Comparison of Experimental and predicted values of pIC_50_ for test set by SMLR, PLS and GA-PLS models.

No.	pIC_50_ (Exp.)	PLS		GA-PLS		SMLR	
		pIC_50_ (Pred.)	Residual	pIC_50_ (Pred.)	Residual	pIC_50_ (Pred.)	Residual
10	7.24	7.34	0.10	6.79	−0.45	7.42	0.18
12	6.50	6.32	−0.17	6.71	0.22	6.35	−0.14
17	7.50	7.44	−0.06	7.82	0.32	7.26	−0.24
2	7.20	7.80	0.60	8.31	1.11	7.67	0.47
21	6.34	6.64	0.30	6.67	0.33	6.68	0.35
25	6.00	6.51	0.51	6.52	0.52	6.10	0.10
25a	8.70	7.81	−0.89	8.72	0.02	7.85	−0.84
37b	6.58	6.46	−0.13	6.57	−0.01	6.16	−0.43
40	5.70	5.73	0.03	6.00	0.30	5.28	−0.42
43	6.00	5.52	−0.48	5.78	−0.22	5.65	−0.35
45b	5.96	5.22	−0.73	5.60	−0.36	6.55	0.59
47	6.14	6.80	0.62	6.70	0.52	5.60	−0.57
51	6.45	6.58	0.12	6.30	−0.15	6.10	−0.35
53b	6.85	6.45	−0.41	6.61	−0.24	6.30	−0.56
58c	8.39	7.60	−0.79	7.31	−1.08	7.67	−0.71
6	7.92	8.50	0.58	7.64	−0.28	8.21	0.29

**Table 13:** Physcicochemical, topological and structural descriptor.

ID	Definition	Group
1	RBN, RBF	Constitutional
2	D/D, J, MAXDN, MAXDP, X5, X0v, X1v, X3v, X4Av, X5Av, X0sol, X0sol, X1sol, X2sol, X3sol, X4sol, X5sol, S0K, S1K, IDDE, IVDE, SIC0, CIC0, IC1, SIC1, CIC1,IC2, BIC4, BIC5, D/Dr05, D/dr06, T(N..O), T(N..S), T(O..O)	Topological
3	BEHm1, BEHm2, BEHm3, BEHm4, BEHm5, BEHm6, BEHv6, BEHv7, BEHe3, BEHe4, BELe5, BELe6	BUCUT
4	GGI2,GGI3,GGI10, JGI1	Galvez topol. Charge indices
5	ATS8m, ATS8v, MATS5e, MTAS6e, GATS4e, GATS5e	2D Autocorrelations
6	qnmax, Qpos	Charge descriptors
7	FDI, PJI3, DISPv, QYYv	Geometrical
8	RDF06u, RDF065u, RDF120u, RDF125u, RDF130u, RDF135u, RDF030m, RDF035m, RDF080m, RDF085m, RDF120m, RDF125m, RDF105v, RDF110v	RDF
9	Mor17u, Mor18u, Mor29u, Mor30u, Mor08m, Mor09m, Mor14m, Mor15m, Mor22m, Mor23m, Mor24m, Mor25m, Mor30m, Mor31m, Mor17v, Mor18v, Mor19v, Mor20v, Mor21v, Mor22v, Mor27v, Mor28v, Mor18e, Mor28e, Mor11p, Mor12p	3D-MoRSE
10	E2u, E3u, E3e, G1p, G2p, E1p, L2s, L3s, G1s, G2s, Au, Am	WHIM
11	HIC, HGM, H3u, H4u, H3m, H4m, H7m, H8m, HATS2m, HATS3m, HATS1e, HATS2e, HATS7p, HATS8p, RARS, REIG, R5u, R6u, R3u+, R4u+, RTu+, R2m, RTm, R1m+, R8m+, RTm+, R1v, R2v, RTv, R1v+, R2e, R3e, RTp,R1p+	GETAWAY
12	MR, PSA, MLOGP	Properties

* Description of descriptors refers to [30].

**Table 14:** Parameters of genetic algorithm GA.

Cross validation	Random subset
Number of subset	4
Window width	2
Initial term %	20%
Maximum generation	100
Convergence (%)	80
Cross-over	Double

**Table 15:**
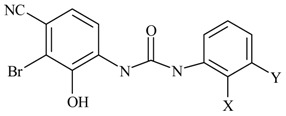
Structural modification of CXCR2 receptor antagonists and predicted activities.

ID	X	Y	GA-PLS (pIC_50_ predicted)	Leverage-limit
1c	H	Br	7.10	0.07
2c	H	Cl	5.63	0.05
3c	H	NO_2_	6.17	0.05
4c	H	OMe	6.01	0.04
5c	H	Me	5.50	0.03
6c	H	Et	5.50	0.04
7c	Br	NO_2_	5.48	0.04
8c	Br	Me	7.20	0.05
9c	Br	OMe	6.67	0.04
10c	Br	Et	8.50	0.06
11c	H	H	6.49	0.04

**Table 16:**
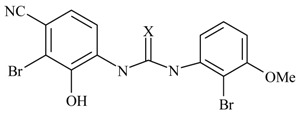
Structural modification of CXCR2 receptor antagonists and predicted activities.

ID	X	GA-PLS (pIC_50_ predicted)	Leverage-limit
10c	O	8.50	0.04
2d	NH	7.74	0.07
3d	NMe	8.82	0.05
4d	NOH	7.91	0.07
5d	NOMe	8.42	0.06
6d	NNH_2_	7.99	0.06
7d	NNHMe	8.39	0.05
8d	NNMe_2_	8.10	0.08
9d	S	8.98	0.05
